# Increasing incidence of DM type 1 in Indonesia

**DOI:** 10.1186/1687-9856-2013-S1-O12

**Published:** 2013-10-03

**Authors:** Aman Pulungan

**Affiliations:** 1 Indonesian Pediatric Society, Dept.of Child Heatlh, Faculty of Medicine,University of Indonesia

## 

Diabetes Mellitus (DM) is one of the most frequent chronic diseases affecting children and adolescents. The number of children being diagnosed with diabetes - regardless to the type of diabetes - is increasing worldwide. It has become a major health problem in both developed and developing countries. The World Health Organization (WHO) data show a prominent worldwide variation in incidence of T1DM from 0.6 per 100,000 in Korea and Mexico to 35.3 per 100,000 in Finland. In Asia, the incidence of type 1 DM is extremely low, from 0.1, 0.6, and 2.4 per 100000 people per year in China, Korea and Japan. Besides type 1 DM, the incidence of type 2 diabetes among children and adolescent is also increasing worldwide. This can be a result of increasing obesity in these population as recent evidences show a strong relationship between childhood obesity and the development of insulin resistance in early adulthood.

Indonesia is the world’s largest archipelago consist of more than 18.000 island. Despite the huge child population in Indonesia which reaches more than 83 million, prevalence of diabetes in Indonesia is still unknown. Very small number of identified cases found through hospital records has not revealed the real burden of this disease as increased cases were found throughout the years. Indonesian Pediatric Society found 825 type 1 DM children from their registration program all over Indonesia during 41 months registry period (February 2009-July 2012, with diagnosis period from 1991 to 2012). Based on our registry data, overall incidence rate of type 1 DM in 2000 was 0,00388 per 100,000 population with 0,00292 per 100,000 male population and 0,00483 per 100,000 female population. In 2010, the overall incidence rate increased to 0,02819 per 100,000 with 0,03884 per 100,000 male population and 0,01761 per 100,000 female population. This increasing can be a result of increased awareness of type 1 DM among health providers and general public, therefore there is improvement in capacity of detecting and managing type 1 DM in Indonesia.

